# Viral Infections of the Central Nervous System in Children: A Systematic Review

**DOI:** 10.7759/cureus.11174

**Published:** 2020-10-26

**Authors:** Abba Musa Abdullahi, Shah T Sarmast, Nusrat Jahan

**Affiliations:** 1 Neurology, California Institute of Behavioral Neurosciences & Psychology, Fairfield, USA; 2 Internal Medicine, California Institute of Behavioral Neurosciences & Psychology, Fairfield, USA

**Keywords:** virus, meningitis, encephalitis, meningoencephalitis, neuroimaging, infants

## Abstract

Viral infections of the central nervous system such as meningitis, encephalitis or meningoencephalitis, are important causes of significant morbidities and mortality worldwide. Early diagnosis and prompt treatment will lead to better outcomes, but any delay may results in high fatality with serious neurologic sequelae among survivors. We conducted a systematic review of published literature on the clinical presentation, diagnosis, treatment and complications of viral infections of the central nervous system from 1980 to 2019 on four databases comprising of PubMed, PubMed Central, Google Scholar and Medline to give the current understanding for better patient management. This systematic review demonstrates the management approach of viral infections of the central nervous system in children from the point of clinical presentation, diagnosis, treatment and complications. Definitive treatment remained unknown; however, certain antiviral drugs were proved to be effective. Therefore, prevention through childhood vaccination is the best management option.

## Introduction and background

Viral meningoencephalitis is one of the emerging debilitating diseases in this century with severe morbidity and mortality as well as difficulty in treating the patients involved. During the last decade, the rate of hospital admissions in England among children from viral meningoencephalitis has increased each year. The incidence of viral meningitis was about 70/100,000 in 2011, and the highest reported incidence was among infants [1]. Viral infections commonly affect infants and immunocompromised individuals who often associated with central nervous system (CNS) involvement. The typical clinical presentations of viral CNS infections are encephalitis, meningitis, myelitis and rarely meningoencephalitis [2]. The spectrum of these clinical presentations depends largely on the viral agents, for instance, arboviruses and some Herpes family viruses like Varicella-zoster virus (VZV) affect brain parenchyma alone or in association with meningis but rarely meningis alone. Thus, they commonly present with encephalitis or meningoencephalitis but rarely meningitis, implying that some viruses cause solely meningitis, some only encephalitis with some others more commonly causing meningoencephalitis [2,3,4].

There are many viruses that are increasingly identified as causes of viral CNS infections, called neurotropic viruses, most of which belong to one of the following five classes of viruses: Picornaviruses, Arboviruses, Paramixoviruses, Arenaviruses (Lymphocytic Choriomeningitis Viruses) and Herpes family viruses [3]. There is a paucity of information regarding the management of viral CNS infections, especially in infants, and the clinical burden of these infections is poorly defined. Moreover, differentiating viral meningitis and meningoencephalitis from other causes of meningitis is very difficult in infants as they usually present with non-specific signs and symptoms mimicking clinical features of other common causes of CNS infections such as bacterial meningitis. This systematic review provides a clinical overview on the presentation, diagnosis, treatment and complications of viral meningoencephalitis in infants to enable proper differentiation of viral etiology from other causes which will significantly reduce avoidable complications, antimicrobial resistance, prolong hospital stay and hospital costs. However, no discussion will be made on encephalitis caused by Rabies and Polio viruses as separate study specifically on them is ongoing by the team members.

## Review

Method

Study Protocol

The Preferred Reporting Items for Systematic Reviews and Meta-Analyses (PRISMA) guidelines were employed during the study.

Source of Data Collection

A multifaceted systematic search of four databases was conducted where published data from 1980 to 2019 were searched for identification of relevant articles. The databases searched were PubMed, PubMed Central, Google Scholar and Medline. A medical subject headings (MeSH) strategy was used for data collection in PubMed and Medline, while regular keyword search was used in other databases.

Inclusion and Exclusion Criteria

Following inclusion criteria were applied for data collection: 1) studies done in the last 40 years with an emphasis on the most recent studies 2) studies exclusively done on infants, infants and other children or infants and other age groups 3) studies done only on humans 4) studies that explained clinical presentation, diagnosis, treatment or complication of viral meningitis or encephalitis 5) studies done globally 7) studies done in English or translated into English.

Search Content

The keywords terms used for identification of articles were “viral meningitis OR encephalitis OR meningoencephalitis” AND “children”, “clinical presentation” AND “viral meningoencephalitis” AND “children”, “diagnosis” AND “viral meningoencephalitis” AND “children”, “treatment” AND “viral meningoencephalitis” AND “children”, “complication OR prognosis” AND “viral meningoencephalitis” AND “children”. Contents of the articles were reviewed, and relevant articles were selected.

Ethical Issue

The purpose of the paper is a systematic review of published studies; therefore, data were collected systematically, accurately and adequately.

Quality Assessment

The assessment tools used for evaluating the quality of included articles were: revised scale for the assessment of non-systematic review articles (SANRA) scale for assessing narrative reviews for the included review articles, Newcastle Ottawa scale for the included retrospective studies and Standardized Case Report Critical Appraisal Sheet for the included case reports and case series. Low-quality papers were excluded, and only high and moderate-quality papers were included in the study.

Results

Literature Search

**Figure 1:**
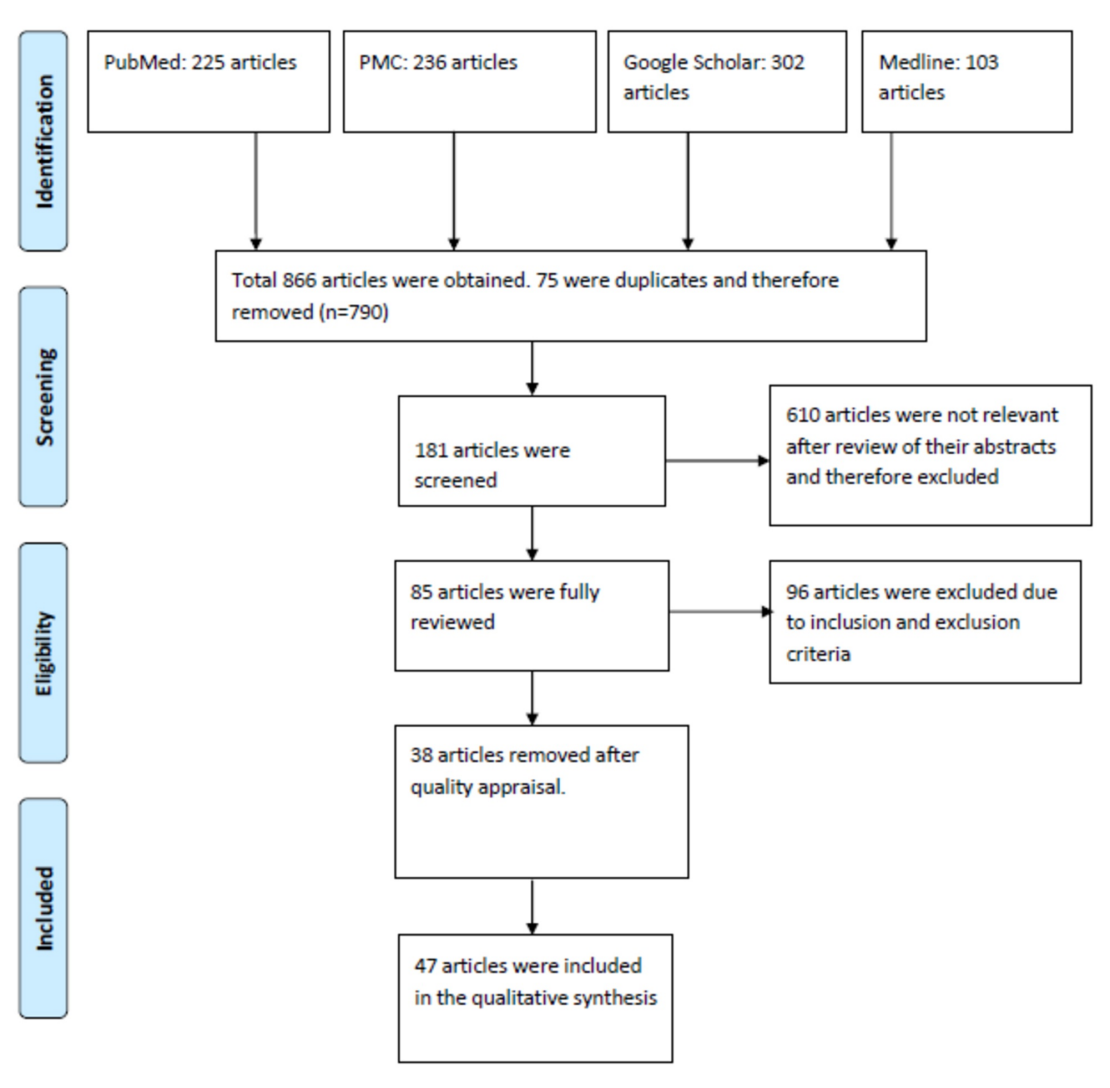
Flowchart of the study selection

After using keywords, a total of 866 articles were obtained: 225 articles from Pubmed, 236 from Pubmed central, 302 from google scholar and 103 from Medline. Six hundred ten articles were found not to be relevant to the study and thus excluded, and 256 articles were selected for assessment. Seventy-five articles were duplicates and therefore excluded, and 181 full-text articles were reviewed for possible inclusion. After applying our inclusion and exclusion criteria, 96 articles were removed, and 85 were fully reviewed. Following the quality appraisal, another 38 articles were further removed, and 47 articles were included in the study. This is depicted in Fig 1 below.

Study Characteristics

Out of the forty-seven included articles, eleven were obtained from Picornaviruses search keywords, eight from Arboviruses, eight from Paramixoviruses, six from Lymphocytic Choriomeningitis Viruses and fourteen from Herpes family viruses search keywords as shown in Table 1 below. Headings were used for the various viral families to achieve the purpose of the study, which is the clinical presentation, diagnosis, treatment and complication or prognosis. Additionally, 18 articles were highly qualitative review articles, 16 articles were retrospective studies, and 13 articles were case-control studies.

**Table 1:** Showing Data Characteristics and Included Studies

Sub-headings	Total study included	Study included From PubMed	Study included From PMC	Study included from Google Sholar	Medline
Picornaviruses	11	5	3	1	2
Arboviruses	8	3	2		3
Paramixoviruses	8	3	1	3	1
Lymphocytic Choriomeningitis Viruses	6	2	2	2	
Herpes Family Viruses	14	5	3	4	2
Total:	47	18	11	10	8

The Study Population Characteristics

The age distribution of the study population was in three categories: infants only, infants and older children and lastly infants and other age groups. Nineteen studies are exclusively for infants, eight studies for both infants and older children and 20 studies for both infants and other age groups. The sex distribution is that the study has a male preponderance.

Picornaviruses

Clinical presentation: The neurotropic members of Picornaviruses are Human enteroviruses (HEVs) and Human parechoviruses (HPeVs) [5]. The common neurologic manifestation cause by HEVs is aseptic meningitis with occasional life-threatening encephalitis in which infants present with fever, irritability, anorexia, shock, limb stiffness and seizures [6,7]. In neonates, the typical clinical presentation is fever and bulging anterior fontanelle [8]. HPeVs CNS infection commonly manifests as meningitis and infants clinically present with fever, poor feeding, irritability, body weakness, loss of consciousness or circulatory shock [1].

Diagnosis: Picornavirus meningitis can be diagnosed by viral cultures of cerebrospinal fluids (CSF), blood, urine or stool or by polymerase chain reaction (PCR) which has higher sensitivity especially when a stool sample is used [9]. Both HEVs and HPeVs have elevated CSF protein and low or normal C-reactive protein, and both can be associated with abnormal brain imaging [1,6]. Reverse-transcriptase real-time quantitative PCR (RT-qPCR) is more sensitive than viral culture and has become the gold standard for diagnosing both HEV and HPeV infections [5].

Treatment: Currently, no definitive antiviral drug for the treatment of picornaviruses and therefore, supportive care is the mainstay of the management. However, it can be well treated with different drugs which were shown to have a very high success rate and these include intravenous immunoglobulin and pleconaril [5,10]. Additionally, intravenous acyclovir and the protease inhibitor, rupintrivir were also reported to have a success rate in the treatment of picornaviruses infection [1,6,11]. The supportive care includes pain control with narcotics, maintaining fluid and electrolyte balance, controlling seizures with antiepileptic drugs like phenytoin and phenobarbitone [5].

Complications: Many studies have reported significant long-term neurologic sequelae in infants with picornavirus CNS infection [5,9] which include status seizure, chronic myoclonic jerks, poor cognitive function with associated abnormal behaviour, delayed developmental milestone and autonomic dysfunction [1,12]. Psychiatric disorders like schizophrenia and attention deficit hyperactivity disorder (ADHD) were reported in children with enterovirus encephalitis [11,13].

Arboviruses

Clinical presentation: Arboviruses cause a wide range of CNS diseases ranging from meningitis, encephalitis, myelitis, encephalomyelitis, neuritis and meningoencephalitis. However, encephalitis is the most common neurologic manifestation. Early symptoms in children include cough, catarrh (flu-like), fever and sore throat and then followed by nausea, vomiting and meningismus [3,4]. There are four main classes of encephalitic arboviruses:

Togaviridae/Alphaviruses

They have a short incubation period of 2-4 days with acute phase lasting 1-2 days which is characterized by fever, sore throat and body pain. It is then followed by anorexia and extreme body weakness which typically last for 2-3 weeks with progression to encephalitis in a small percentage of cases (0.05-4%) [4]. The common members of this class include Eastern, Western and Venezuelan Equine Encephalitis viruses (EEEV, WEEV and VEEV respectively) which clinically present with fever, convulsion, neck stiffness or coma [4,14]. Another member is Chikungunya virus (CHIKV) which presents with fever, convulsion and features of multi-organ damage [15,16].

Flaviviridae

Members of this class commonly present with meningitis or meningoencephalitis characterized by recurrent fever, body stiffness, body weakness, convulsions and signs of meningeal irritation [17,18]. They include Japanese encephalitis virus (JEV), tick-borne encephalitis virus (TBEV), Powassan encephalitis virus (POWV), Dengue virus (DENV), Yellow fever virus (YFV), West Nile virus(WNV), St. Louis encephalitis virus (SLE) and Zika virus (ZIKV) which commonly occur as congenital infection clinically manifested with microcephaly and features of cognitive impairment [18].

Bunyaviridae

The neurotropic members include California encephalitis virus and La Crosse encephalitis virus (CEV and LACV respectively) which commonly present as encephalitis characterized by fever, nausea, vomiting, abdominal distention, lethargy, convulsions and loss of consciousness with an incubation period of 3-7 and 5-15 days respectively. Other members are Rift Valley Fever virus (RVFV) and Toscana virus (TOSV) which are mostly asymptomatic, but when symptoms usually occur present with meningitis or meningoencephalitis [4].

Reoviridae

The clinically significant member of this class is Colorado Tick Fever virus (CTFV) which clinically presents with fever, nausea, vomiting, abdominal distension, skin rashes and photophobia which is then followed by features of meningitis or meningoencephalitis [4].

Diagnosis: All arboviruses can be diagnosed by a single RT-PCR protocol and multiple primer pairs. Enzyme-linked immunosorbent assay (ELISA) has also been used to successfully diagnosed arboviruses by detecting arbovirus specific immunoglobulin M antibody [15]. Neuroimaging is another essential tool for confirming a diagnosis and for determining the extent of brain damage. Both computed tomography (CT) scan and magnetic resonance imaging (MRI) are used, but MRI tends to be more diagnostic [16,19]. RT-PCR can be done on CSF, blood, urine or breast milk [3,20]. Diagnosis is also supported by CSF pleocytosis and high proteins [16,17].

Treatment: Also, no definitive therapy for the treatment of encephalitic arboviruses and therefore, management is mainly supportive [4,17]. Some arboviruses like ZIKV and CHIKV, however, demonstrated a remarkable improvement when treated with intravenous immunoglobulin, plasmapheresis and corticosteroid [20]. The supportive care includes the use of antipyretic for fever, correcting dehydration in cases of severe emesis, or use of life support to maintain respiratory and circulatory systems in patients requiring intensive care unit (ICU) admission and seizure control with anticonvulsants like fosphenytoin and levetiracetam [3,4,17]. A purine analogue called Favipiravir was shown to be very effective with a promising result for many arboviruses such as bunyaviruses, flaviviruses, and alphaviruses [21].

Complications: Worldwide, encephalitic arboviruses cause life-long neurologic illness at the rate of 50 to 100,000 cases/year with neurocognitive sequelae in survivors such as deafness, mental retardation, emotional liability and hemiparesis [4,17].

Paramyxoviruses

Clinical presentation: Infection with Paramixoviruses causes a wide range of CNS manifestations from meningitis to meningoencephalitis and less common but fatal encephalitis [3]. These viruses include Mumps virus (MuV), Measles virus (MV) and Henipaviruses comprising of Nipah and Hendra viruses (NiV and HeV respectively).

MuV can manifest as asymptomatic aseptic meningitis or more commonly as symptomatic aseptic meningitis which is characterized by fever, nausea, vomiting, headache (in older children), neck stiffness and meningismus. The less common but severe manifestation is encephalitis which occurs in less than 0.5% of cases of mump infection and characterized by fever, focal or generalized convulsions, signs of meningeal irritation, delirium and coma. Meningoencephalitis can occur concomitantly with meningitis which is both benign and self-limiting with no serious complications [3,22,23].

The neurologic manifestation of MV ranges from Acute demyelinating encephalomyelitis (ADME) which is an acute inflammation of the brain with associated myelin damage, Measles inclusion body encephalitis (MIBE) which occurs in patients with depressed immunity and Sub-acute sclerosing panencephalitis (SSPE) which occurs as a complication about 4years after MV infection and therefore less common in infants. The signs and symptoms include febrile convulsions, poor appetite, signs of meningeal irritation or unconsciousness [21,24].

After a prodromal phase of fever and lethargy, Henipavirus infection results in relapsing encephalitis which is characterized by febrile convulsions, limb weakness, segmental myoclonus, dizziness, vomiting, impairment in spatial perception, altered consciousness, drowsiness, and abnormal plantar response which rapidly progressed to coma within 24-48 hours [25].

Diagnosis: MuV CNS infection can be diagnosed by CSF pleocytosis with normal or slightly elevated CSF protein. Definitive diagnosis can be achieved by viral cultures of buccal mucosal swab or CSF, RT-PCR or an ELISA assay to detect mumps virus antigen (IgM or IgG) in CSF [26].

MV infection can be diagnosed by assessing measles antigen, IgG or IgM using an automated qualitative enzyme-linked fluorescent immunoassay and a quantitative indirect immunofluorescence test. RT-PCR can also be used to confirm the diagnosis by detecting viral RNA [24,27]. Viral cultures of CSF, blood, urine and conjunctival swab has become an essential tool for making the definitive diagnosis [21].

The diagnosis of Henipavirus infection is by CSF examination, which may show elevated blood cell count and protein. Computed Tomography (CT) scan can be normal, but Magnetic Resonance Imaging (MRI) is almost always pathologic. Definitive diagnosis is achieved by ELISA, PCR assay, serum neutralization, immunofluorescence assay and virus isolation by cell cultures using blood, urine, CSF and throat swab [25].

Treatment: Currently, the mainstay of management is symptomatic treatment as no definitive antiviral drug for treating paramyxovirus neurologic diseases [21,25]. Ribavirin, which has broad-spectrum antiviral activity against both RNA and DNA virus, was shown to be an effective drug for the management of encephalitis in both MV and NiV infections. Favipiravir and Balapiravir were also found to be active against NiV and HeV neurologic diseases in various studies [25].

Complication: Obstructive hydrocephalus and acute axonal polyneuropathy were reported in rare cases of mumps meningitis; however, the recovery is rapid and excellent. Death in mump encephalitis occur in up to10- 20 % of cases, while 33 % of survivors show evidence of prolonged neurological sequelae [26,28]. Subacute sclerosing panencephalitis is a fatal, progressive degenerative central nervous system disease that usually presents 5 to 10 years after the measles virus infection caused by persistent defective measles virus in neurons and oligodendrocytes [21,24].

Arenaviruses (Lymphocytic Choriomeningitis Virus)

Clinical presentation: Lymphocytic Choriomeningitis Virus (LCMV) infection in children usually present with signs of meningitis, and when counteracted post-natally, it is associated with aseptic meningitis which occurs in about 15% of patients with confirmed infections [29]. Rarely meningoencephalitis and encephalitis were reported with severe complications. Cases of congenital microencephaly and teratogenesis caused by LCMV were reported in neonates [3,30,31].

Diagnosis: Definitive diagnosis can be achieved using ELISA and indirect immunofluorescence assay for detecting specific anti-LCMV IgM or IgG antibodies or PCR for detecting viral RNA from CSF or blood [29,32]. Viral culture of CSF can be done only when the infection is acquired postnatally [33].

Treatment: Favipiravir and Rivabirin are the two antiviral drugs that have shown promising results against arenavirus LCMV; however, no specific treatment for the management of LCMV CNS infection, and therefore supportive care remained the treatment modality [32,33,34].

Complications: LCMV congenital infection has a poor prognosis with a high mortality rate of about 35%. The survivors usually have severe neurodebility including microcephaly, mental retardation, hydrocephalus, chorioretinitis, visual impairment and seizure disorder [29,33].

Herpes Family Viruses

Clinical presentation: HSV CNS infection also has a wide range of clinical manifestation comprising of meningitis, encephalitis, and meningoencephalitis. Aseptic meningitis is the most typical CNS manifestation presenting with fever, convulsion, and loss of consciousness. The less common Herpes simplex encephalitis (HSE), present with fever, convulsion, abnormal behaviour, disorientation and neurologic relapse [35,36,37]. In neonates, HSV meningitis usually has non-specific symptoms, including body weakness, irritability, poor oral intake, and fever leading to misdiagnosis and high rate of mortality. Neonatal HSE is the most severe but rare presentation, usually associated with lethargy, fever and convulsions [38]. Early diagnosis is also tricky as initial symptoms are usually non-specific [39,40]. Childhood HSE is predominantly caused by HSV-1, while HSV-2 is commonly isolated in neonates and infants as it is almost always acquired from an infected maternal genital tract [35]. The members of this family include VZV, EBV and CMV.

Varicella-zoster virus (VZV) is a member of herpes viral family, and it is usually associated with meningoencephalitis and postinfectious encephalopathy which present with fever, vomiting, bulging of anterior fontanelle, downward gaze deviation, apathy, convulsions, loss of consciousness and focal neurologic deficit. Aseptic meningitis is rare with VZV infection [41,42]. Another member is Epstein Barr virus (EBV) which commonly cause encephalitis that clinically present with fever, convulsions or loss of consciousness [43]. CMV CNS infection also commonly present with encephalitis that primarily affects immunocompromised patients and the commonest manifestation in infants is congenital infection presenting with fever, convulsions, vomiting, poor appetite, signs of meningeal irritation and altered level of consciousness [44,45].

Diagnosis: Diagnosis of HSV CNS infection is confirmed by one of the following three modalities: 1)viral cultures of conjunctival, nasopharyngeal, mouth or anus swab 2) PCR of CSF or blood for detection of viral DNA. Other supportive diagnostic findings include CSF pleocytosis, mildly to moderately elevated CSF protein, and abnormal neuroimaging using MRI [39,40]. VZV CNS infection is diagnosed using PCR were the viral DNA is identified from CSF, saliva or skin swab. There is also associated with CSF pleocytosis and elevated CSF protein [41,43].

The diagnosis of EBV CNS infection can be confirmed by using PCR for detecting viral DNA in the CSF and serologic or CSF determination of specific antibodies to viral capsid antigen (VCA-IgM). Neuroimaging particularly MRI can reveal a wide range of brain abnormalities. CSF pleocytosis and an elevated CSF protein can also be observed [44]. The definitive diagnosis of CMV CNS infection is achieved by PCR of the CSF and also by detecting anti-CMV IgM, CSF pleocytosis and elevated CSF protein is also supportive [45,46].

Treatment: Intravenous acyclovir is currently the recommended drug for the treatment of HSV CNS infection in neonate and infant likely due to its minimal side effects. However, neonatal HSE can be well treated with Idoxirudine and Vidarabine which significantly reduced morbidity and mortality but has limited tolerability than acyclovir and treatment should be commenced early within two days of onset of neurological symptoms [36,37,47]. The recommended dose of acyclovir for the management is 10mg/kg/dose over 1hr as infusion 8hrly for 10-14days; however, some studies suggested high dose of acyclovir of 60mg/kg/day in 3 divided doses eight hourly but safety cannot be ascertained [3,36,37]. Other acyclic nucleoside analogues such as valacyclovir and famciclovir have higher bioavailability than acyclovir and thus are used as oral regimens. The supportive measures for HSE include seizure control with anticonvulsant, adequate hydration as high-dose aciclovir can cause acute renal failure and adjunctive steroids [36,37,47].

The treatment modality for VZV encephalitis is with high dose intravenous acyclovir 10-20mg/kg/dose 8hrly for 14days and one-week course of oral corticosteroids like prednisolone at 2 mg/kg/day maximum. Oral drugs like valaciclovir and famciclovir can be used instead of acyclovir [43]. No definitive drug for the treatment of EBV neurologic diseases; however, some studies showed beneficial effects of acyclovir, valacyclovir, ganciclovir and valganciclovir with high success rate. Other supportive measures include use of antipyretics for fever, pain control with analgesic, maintenance of fluid and electrolytes, adequate nutrition and bed rest [44]. CMV encephalitis can be effectively treated with nucleosides like acyclovir, penciclovir, ganciclovir, and its oral prodrugs like valacyclovir, famciclovir, and valganciclovir. Nucleotides like Cidofovir and pyrophosphates like foscavir are currently approved for the treatment of human cytomegalovirus encephalitis [45,46].

Complications: About 70% of untreated HSE lead to death with a permanent neurologic deficit in almost all survivors including microcephaly, hydrocephaly, porencephalic cysts, spasticity, chorioretinitis, blindness, deafness, learning disabilities and seizures [3,37]. Congenital CMV is considered as the leading cause of non-genetic cause of sensorineural hearing loss (SNHL) in children and an important cause of neurodevelopmental delay [46].

Discussion

Viral CNS infections from many viruses of different viral families cause a wide range of clinical spectrum in humans from meningitis, encephalitis or even serious meningoencephalitis. The most vulnerable population are infants and immunocompromised. In this systematic review, we have provided an approach on how to manage these infections clinically from the point of identification, diagnosis and treatment. It can be understood from the study that viral infections of the central nervous system should be suspected in any pediatric population, especially infants presenting with neurologic signs and symptoms and should always be a differential of bacterial meningitis. It is also pointed out in the study that definitive treatment of viral CNS infection is currently not available; however, reported studies showed the beneficial effects of specific antiviral drugs like acyclovir or ribavirin if coupled with proper supportive management. Therefore, prevention through childhood vaccination is the most effective management approach to these infections.

This current study is significant scientifically and clinically. Scientifically, the paper will help the scientific community and future researchers as an exhaustive search was conducted via major databases in an attempt to gather all the available information on viral meningoencephalitis and other clinical spectra in infants. Clinically, the paper has provided a holistic approach on the management of almost all neurotropic viruses in infants from clinical presentation to diagnosis, treatment and complications. This understanding will help not only the clinician in making a prompt diagnosis and instituting early management but also the patient through preventing complications and minimizing hospital stay and cost.

The study also has some limitations, which include restricting the study only to viral etiology without considering other causes. Also, it is limited only to the clinical presentation, diagnosis, treatment and complications without discussing the virology, epidemiology and pathogenesis. Another limitation is the focus given only to papers published in the past 40yrs with emphases on the most recent studies as the majority of the studies were from 2010 to 2019. Future research is required, especially on identifying the definitive antiviral drug for the different neurotropic viruses. Also, prospective studies should be conducted with the aim of further delineating clinical features and diagnostic tests of viral CNS infections.

## Conclusions

Viruses are among the leading cause of meningitis, encephalitis or meningoencephalitis worldwide with serious and devastating consequences. There are many classes of viruses that can cause central nervous system infections belonging to different viral families. The most vulnerable population for viral infections are children, especially infants and immunocompromised. However, little has been done on the clinical course of viral infections of the central nervous systems in children, leading to delay in diagnosis and subsequently debilitating consequences. Therefore, this systematic review is an attempt to explain the clinical presentation of viral meningoencephalitis, meningitis and encephalitis in a pediatric population with more emphasis on infants, the different viral etiologies that should be suspected, the diagnostic and the treatment approach. Additionally, the complications of viral infections of the central nervous system in children were fully elucidated so that appropriate rehabilitative therapies would be commenced. It is clear from the study that the definitive treatment could not be ascertained, but certain antiviral drugs were proved to be effective. Therefore more researches are needed in future, which will pave ways for developing a standard antiviral regimen for the treatment of the viral central nervous system infections.
